# On modeling locus heterogeneity using mixture distributions

**DOI:** 10.1186/1471-2156-5-29

**Published:** 2004-09-30

**Authors:** Shili Lin, Swati Biswas

**Affiliations:** 1 Department of Statistics, The Ohio State University, Columbus, OH 43210, USA; 2 Department of Biostatistics, The University of Texas MD Anderson Cancer Center, Houston, TX 77030, USA

## Abstract

**Background:**

Locus heterogeneity poses a major difficulty in mapping genes that influence complex genetic traits. A widely used approach to deal with this problem involves modeling linkage data in terms of finite mixture distributions. In its simplest setup, also known as the admixture approach, a single parameter is used to model the probability that the disease-causing gene of a family is linked to a reference marker. This parameter is usually interpreted as the overall proportion of linked families.

**Results:**

In this article, we address two issues regarding the admixture approach. First, we tackle the question of whether the single parameter of linked proportion is well defined in general. By formulating the likelihood under a classification scheme based on distributions, we show that such a parameter is meaningful only when a certain well-characterized condition is met. Second, we study a condition given in the literature for validating the admixture approach. A counter example is constructed to illustrate that the condition does not necessarily lead to valid estimates.

**Conclusions:**

Estimators from the admixture approach may be inconsistent. This holds even if a condition given in the literature to validate the approach is satisfied.

## Background

Mapping disease genes influencing complex genetic traits is far more difficult than mapping genes underlying Mendelian traits. One of the difficulties is due to locus heterogeneity, in which the disease in different families (or in different individuals within a family) is caused by different loci or non-hereditary factors. Finite mixture distributions have been proposed to model linkage data in the presence of locus heterogeneity [1], also known as admixture modeling. It is currently a widely used approach for testing heterogeneity and/or linkage [2]. In its simplest setup with two parameters, each independent data unit (usually a single family) is assumed to be either linked or unlinked. Here a family is said to be linked if its disease-causing gene is linked to a reference marker. In the two-parameter setup, all linked families are assumed to have the same linkage parameter, the recombination fraction *θ*. The heterogeneity is modeled by a single parameter (*α*) that denotes the probability that a family is linked. This parameter is interpreted by many researchers as the overall proportion of linked families.

Despite the popularity of the admixture approach, a number of authors have pointed out limitations with the approach, albeit reaching different conclusions on its practical values. For example, one of the assumptions of the admixture approach is that there is only inter-family, but no intra-family, heterogeneity. Goldin [3] and Durner et al. [4] carried out simulation studies to show that violation of this assumption does not necessarily lead to a loss of power for detecting linkage. Another inherent assumption of admixture approach is that the genetic models at the linked and unlinked loci are the same. Vieland and Logue [5] showed that violation of this assumption leads to asymptotically biased parameter estimates. They showed that in this situation, the parameter *α *does not even measure the proportion of linked families within the sample, contrary to popular belief. Similar conclusions were obtained in the simulation study by Pal and Greenberg [6], in which the authors simulated data under various two-locus heterogeneity models, genetic parameters, ascertainment schemes, and phenocopy frequencies to see their effect on *α*. Nevertheless, these studies as well as some other simulation studies (see [7] and references therein) support the use of admixture approach as a robust tool for testing linkage in the presence of heterogeneity.

Other researchers take a different view, pointing to potentially severe biases of the estimates obtained from the admixture approach (e.g., [8,9]). Whittemore and Halpern [9] provided comprehensive discussion on the genetic assumptions underlying the admixture approach. One of the foci was on whether the admixture parameter (*α*) is meaningful when certain assumptions are violated. Janssen et al. [8] showed, through examples, that estimates of *α *and *θ *can be severely biased when the distribution of *informativeness *in the linked families is not roughly the same as that in the unlinked families, under some measure of informativeness for linkage studies. They used a measure called Effective Number of Informative Meioses (EFNIM) to assess the informativeness of a family. It is based on the Expected LOD (ELOD) score of a family. They argued that, if the Linked and Unlinked Families are nearly Equally Distributed (LUFED) in terms of EFNIM, then the admixture procedure should provide satisfactory, i.e., nearly unbiased, results.

In this article, we consider the same problems discussed in Janssen et al. [8] and Whittemore and Halpern [9], but from a more statistical perspective in terms of formulating the likelihood based on finite mixture distributions. In the usual formulation of finite mixture modeling for statistical inference, the data in the sample are independent and identically distributed (iid). The distribution of each of these data points is a finite mixture of several component distributions, with known or unknown mixing proportions, called weights [10,11]. In linkage analysis with family data, unless all families follow the same distributions both under linkage and no linkage (i.e., all families having the same component distributions), the usual admixture approach does not reflect the correct likelihood function (or, equivalently, the heterogeneity LOD function). In most genetic studies using family data, this condition is violated as data usually come from families of various structures, sizes and complexities. That is, the families are not iid; they are independent but not necessarily identically distributed. Therefore, the admixture modeling with a single heterogeneity parameter is under parameterized. So, a natural question is what is its implication on the estimators of the parameters. Are they consistent? Under what condition does consistency hold? We investigate these questions by formulating a more general likelihood that assigns a separate heterogeneity parameter to each class of families following the same distribution. Then using this formulation, we show that the estimators from the admixture formulation are consistent only if a certain condition holds. Furthermore, we study the argument by Janssen et al. [8] on the LUFED condition for obtaining nearly unbiased estimates. We show that LUFED is a necessary condition for obtaining consistent estimates. However, as we illustrate through counter examples, this condition is not sufficient to guarantee consistency. In fact, for certain data satisfying LUFED, the asymptotic estimates can be far from their true values.

## Results

### Formulation of expected log-likelihood

Suppose that data from independent families in the sample are classified into *T *types such that families in the same type follow the same distribution. That is, families are classified into the same type based on their "structures" that lead to the specification of their distributions. Here we use the term structure in a broad sense that includes not only the pedigree structure but also any other available information about the pedigree (such as knowledge of phase) that contributes to the distribution from which the family is generated. Note that structure does not include any phenotypic or genotypic data of a family. Families with the same structure but different phenotypic and/or genotypic data are simply different realizations from the same distribution and hence are iid, and are classified into the same type. In the most general setup, with *g *potential linkage scenarios, e.g., linked or unlinked (*g *= 2) in the simplest setting, the probability distribution, *f*_t*_, for a realization (*y*_*t*_) from type *t*, *t *= 1,…,*T*, can be expressed in terms of its component distributions, *f*_*ti*_, *i *= 1,…,*g *[10]:



where the mixing parameters satisfy 0 ≤ *α*_*ti *_≤ 1 (*i *= 1,…,*g*), , and ***θ ***is the linkage parameter vector, including various recombination fractions.

To simplify our presentation without compromising our objectives, we assume that the disease under study is caused either by a locus linked to the reference marker with a recombination fraction *θ *or by a locus unlinked to the reference marker. Further, we assume that the genetic (trait) models at the linked and unlinked loci are the same. So, the linkage parameter vector is ***θ ***= {*θ*, 1/2}, and the probability distribution is simplified to

*f*_t*_(*y*_*t*_; {*θ*,1/2}) = *α*_*t*_*f*_*t*_(*y*_*t*_; *θ*) + (1 - *α*_*t*_)*f*_*t*_(*y*_*t*_; 1/2),

where *α*_*t *_is the probability that a type *t *family is linked. Note that the two distributions under the mixture have the same parametric form but differ in their linkage parameter values, being *θ *or 1/2. Also, note that a single *α *parameter is used for all families in the same type.

Let *a *denote the true proportion of linked families in the entire sample. Let *p*_*t*_, *t *= 1,…, *T*, be the true proportion of type *t *families in the linked group. Thus ***p ***= (*p*_1_,…, *p*_*T*_) is the distribution of types among the linked families. Recall that all families in a given type have the same distribution, i.e., same likelihood for any value of the recombination fraction. Similarly, define ***q ***= (*q*_1_,…, *q*_*T*_) to be the distribution of family types among the unlinked group. Then the probability that a family is of type *t *is *s*_*t *_= *ap*_*t *_+ (1 - *a*)*q*_*t*_, and the probability that a type *t *family is linked is thus . Furthermore, let *r *denote the true common recombination fraction for the linked families. The likelihood contribution for estimating the parameters {*α*_*t*_, *θ*} by a type *t *family with data *y*_*t*_ is

*L*_*t*_(*α*_*t*_, *θ *| *y*_*t*_) = *α*_*t*_*f*_*t*_(*y*_*t*_; *θ*) + (1 - *α*_*t*_)*f*_*t*_(*y*_*t*_; 1/2).     (1)

Then the expected contribution to the log-likelihood from a family of type *t *can be expressed as



Note that *r *and  are the true underlying quantities, whereas *θ *and ***α ***= (*α*_1_,…, *α*_*T*_) are the parameters to be estimated from the data. Combining the expected log-likelihoods, ELL_*t*_, *t *= 1,…, *T*, we can form the total expected log-likelihood from all family types as follows:



Since the above formulation of likelihood is correct under the stated assumptions, it can be shown that ELL(***α***^0^, *r*) ≥ ELL(***α***, *θ*), for any (***α***, *θ*), following Stuart and Ord [12]. This implies that this formulation of likelihood is guaranteed to provide consistent estimators of (***α***, *θ*). Note that ***α ***is a vector of nuisance parameters; only *θ *is the true parameter of interest.

We note that the above likelihood, although similar to the one presented in Vieland and Logue [5], is different from it in two respects: First, their likelihood (and hence log of likelihood ratio, referred to as 2T-HLOD in their paper) allows for different trait models at the linked and unlinked loci (as they wanted to see the effect of incorrectly assuming same trait models at the two loci) while these two are assumed to be the same in (1). Second, 2T-HLOD involves different *α *parameters for families with different structures as well as different phenotypic data. So, families with the same structure have different *α *parameters if they have different phenotypic data. On the other hand, in (1) families with the same structure (and hence their data coming from the same distribution as elucidated at the beginning of this section) share a common *α *parameter. Since the issue addressed by Vieland and Logue [5] is different from ours and is based on different assumptions, the two likelihood formulations are not directly comparable.

#### The two-parameter setup

In this subsection, we show that the two-parameter setup leads to correct likelihood formulation only when ***p ***= ***q***, that is, the distribution of family types among the linked families is the same as that among the unlinked families. In this setup, all *α*_*t*_'s are set to be equal to a common parameter, *α*, the overall proportion of linked families. Hence, the total expected log-likelihood is with respect to the recombination fraction *θ *and a single proportion parameter *α*:



The expected log-likelihood ratio between the two sets of parameter values, (*a*, *r*) and (*α*, *θ*), is



where



When *p*_*t *_= *q*_*t *_for each *t*,



and by Jensen's inequality [13]



In other words, when ***p ***= ***q***, ELL(*a*, *r*) ≥ ELL(*α*, *θ*), for any (*α*, *θ*), implying that  = *a *and  = *r *are the maximum likelihood estimates of *α *and *θ*. However, as shown in the examples in the next section, when ***p ***≠ ***q***, ELL(*a*, *r*) may be smaller than ELL(*α*, *θ*) for some parameter values (*α*, *θ*). That is, the maximum likelihood estimates of *α *and *θ *may not be consistent because the likelihood model is incorrectly specified (under-parameterized). In order for the two-parameter formulation to be correct, the following constraints should be placed on the makeup of the families:



which implies that ***p ***= ***q ***given the additional constraints that  and . Notice that ***p ***= ***q ***is stronger than the LUFED condition. Equal distributions of types in the linked and unlinked families according to the classification scheme based on distributions of the data implies equal distributions of informativeness in the same two groups. Therefore, LUFED is a necessary condition for the admixture approach. We also note that ***p ***= ***q ***is not necessarily satisfied for every dataset (see Discussion).

In general, in a parameterization that demands an estimate for the overall proportion of linked families, *α*, the relationship between *α *and *α*_*t *_is as follows:



where *u*_*t *_and *v*_*t *_are also unknown parameters (with constraints ∑*u*_*t *_= 1 and ∑*v*_*t *_= 1) that need to be estimated. Note that *u*_*t *_and *v*_*t *_are the parameters to be estimated from the data and are not necessarily the same as *p*_*t *_and *q*_*t*_, respectively, as the latter are the *true *values of the former. We have shown earlier that the maximum likelihood estimates for *α*_*t *_exist. However, one cannot solve the equations in (3) to obtain a corresponding estimate for *α*. This is because there are only *T *+ 2 equations (including the two constraints) but 2*T *+ 1 unknown parameters, and thus not all parameters are identifiable when *T *≥ 2. Hence, one may not be able to find a meaningful estimate of the overall proportion of linked families, contrary to popular desire for such an estimate. This is consistent with the finding of Whittemore and Halpern [9], although the conclusions are reached from two different perspectives (see Discussion).

### Further investigation of LUFED condition

We have already shown that LUFED is a necessary condition for obtaining consistent estimators using the admixture approach. In this section, we investigate, through a contrived dataset, whether LUFED is also a sufficient condition for achieving satisfactory results, as contended by Janssen et al. [8]. We use ELOD, a popular measure of informativeness [2], for family type classification in terms of their informativeness for linkage studies. This measure is the basis for the EFNIM criterion of Janssen et al. ([14,8]); see these two references for detailed description of EFNIM.

In our contrived dataset, the linked group consists of two types of families: Phase Known (PK) double backcross families in proportion *p*_1_, and Phase Unknown (PU) families in proportion *p*_2_, where *p*_1 _+ *p*_2 _= 1. The unlinked group is also composed of PK and PU families, but in proportions *q*_1 _and *q*_2_, respectively, where *q*_1 _+ *q*_2 _= 1. We choose to work with the PK and PU families as the PK families were used by Janssen et al. [8] to evaluate the admixture approach, and more generally, these family types are frequently used to evaluate exact properties of linkage analysis methods [2]. We assume that there are *m *children in all PK families and *m *+ 1 children in all PU families. As these two types of families have different distributions [2], we have two family types (*T *= 2) under the classification scheme according to distributions.

Now, let us consider the ELOD of each family. The ELOD of a PK family with *m *children is:



where, as before, *θ *is the parameter for recombination fraction while *r *is the true recombination fraction. Similarly, one can find the ELOD of a PU family with *m *+ 1 children:



For *r *close to 0, it can be seen that ELOD_PK_(*θ*) and ELOD_PU_(*θ*) are both approximately *m *log_10 _2, when they are evaluated at *θ *= *r *[2]. Hence, both PK and PU families, irrespective of their linked or unlinked status, are of one common type according to the ELOD criterion for classification. Thus the data satisfy Janssen et al.'s [8] LUFED condition. Note that the ELOD criterion for classification (and hence the LUFED condition) is based on homogeneity LOD scores to evaluate the informativeness of a family for linkage study and thus does not involve *α *parameters.

It is worth noting that this dataset may serve as an example for understanding classification based on distributions and family realizations that make up a family type. The phase information as well as the number of offspring are part of the family structure, which is the basis of classification according to distributions. However, phenotypes and genotypes do not configure into this classification scheme. For example, the PK data type consists of families with several phenotypes, i.e., various combinations of recombinant/non-recombinant (*m*/0, *m *- 1/1,..., 1/*m *- 1, 0/*m*) offspring.

**Figure 1:**
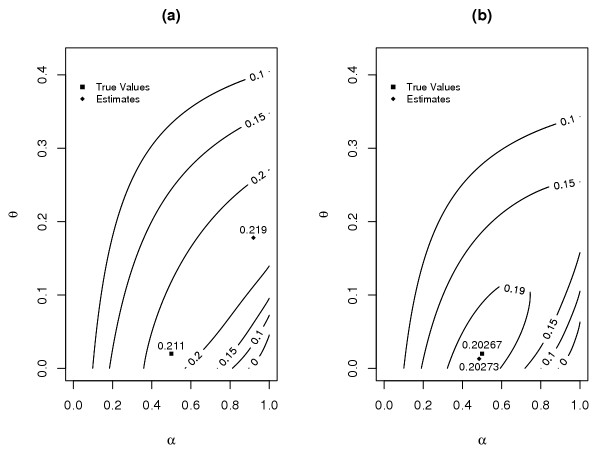
Contour plots of the expected log-likelihood for the two-parameter model: (a) *p*_1 _= 0.9, *p*_2 _= 0.1, *q*_1 _= 0.1, *q*_2 _= 0.9; (b) *p*_1 _= 0.3, *p*_2 _= 0.7, *q*_1 _= 0.4, *q*_2 _= 0.6. The numbers in each plot are various levels of the expected log-likelihood. The expected log-likelihoods at the parameter combinations represented by a curve are the same.

To illustrate our results, we consider two specific settings for the distributions of families in the linked and unlinked groups under the classification scheme according to distributions: (a) ***p ***= (0.9, 0.1), ***q ***= (0.1, 0.9); and (b) ***p ***= (0.3, 0.7), ***q ***= (0.4, 0.6). For each of these two settings, we take *m *= 3 children in PK families and *m *+ 1 = 4 children in PU families. In both settings, the overall proportion of linked families, *a*, is 0.5, and the true (small) recombination fraction, *r*, is 0.02. So, as discussed above, the distribution of informativeness among the linked families, under the ELOD measure, is the same as that among the unlinked families. The two pictures in Figure 1 show the contour plots of the expected log-likelihood under the two settings, for the two-parameter admixture formulation as given in (2). We see that the values of the parameters (*α*, *θ*) where the maximum expected log-likelihood occurs are not the same as their true values. So the estimates are inconsistent. The extent of this inconsistency depends on several factors, including how close ***p ***is to ***q***. As seen in Figure 1 (a), where ***p ***differs greatly from ***q***, the parameter estimates are far away from the true values. On the other hand, in Figure 1 (b), where ***p ***is close to ***q***, the estimates are not too far from the truth. In fact, the estimates are expected to converge to the true parameter values when the difference between ***p ***and ***q ***approaches **0**.

## Discussion

This article addresses two questions. Is the single parameter representing the overall proportion of linked families in the admixture approach well defined? Can one validate the admixture approach if the distributions of linkage information in the linked and unlinked families are roughly the same according to a certain measure of informativeness? A simple situation, where the disease is caused either by a linked or an unlinked locus following the same genetic model, suffices for our purpose. That is, although issues such as age-dependent penetrances, locus-dependent models, and intra-family heterogeneity are very important in analyzing data in the presence of locus heterogeneity, they are not within the scope of this article.

The first question of interest was addressed in Whittemore and Halpern [9], where they characterized the *genetic *conditions under which the parameter for overall linked proportion is meaningful. In contrast, we consider the same question from a more statistical perspective by characterizing *statistical *conditions under which the linked-proportion parameter is well defined and consistent estimate can be obtained. Our basic argument is built upon the realization that an implicit assumption in the usual admixture approach can be badly violated. The admixture approach assumes that, implicitly in the way its likelihood is formulated, all families follow the same distribution. However, data from different families may follow different distributions, defining various types of data. Our results show that, if the distributions of the family types, classified by the distributions of the data, are the same for both the linked and unlinked families, i.e., ***p ***= ***q***, then the parameter is meaningful, and a consistent estimate exists. Otherwise, the desire for an estimate of such a parameter is ill-conceived. Note that the condition ***p ***= ***q ***may not be satisfied in practice, even asymptotically, because linked and unlinked groups may have other differences such as different fertility levels, age of disease onset, disease severity, etc, that may indirectly lead to different family structures (and hence different distributions) in the two groups. Although the problem considered in Vieland and Logue [5] is different from what we consider, as they focus on violation of different implicit assumptions of this approach, from a broader perspective, their as well as our conclusions demonstrate the inconsistency of estimators obtained from the admixture approach.

The second question stems from our curiosity on an argument made by Janssen et al. [8] for the admixture approach. We show, through counter examples with a contrived dataset, that the answer to the question is *no *under the ELOD measure for informativeness. The difference between the estimates and the true values of the parameters, even with infinite amount of data, can be large, if one would carry out the analysis under the admixture approach. This should serve as a warning against complacency when the LUFED condition is met. We do realize that, with phase known and phase unknown data, these examples can be quite extreme in human genetic studies, although such data arise frequently in experimental crosses. In situations where data from more general family structures are available, the effect of violation of the admixture approach assumptions may be much smaller.

The criteria for classification of families deserve further clarification because it is the heart of the problem. We discuss two criteria for classification, one according to the distributions of the data, and the other according to a measure of informativeness of the data for linkage studies (leading to the LUFED condition). The classification scheme based on the distribution criterion leads to a necessary and sufficient condition for validating the admixture approach. This result is a by-product of our theoretical development for finding an answer to the first question. The classification scheme based on the informativeness criterion, on the other hand, leads to the conclusion that LUFED is only a necessary, but not a sufficient, condition for the admixture approach, contrary to Janssen et al.'s [8] contention.

Finally, we note that although the general formulation of likelihood based on forming groups of families with the same distribution gives consistent estimators, its practical utility seems to be limited. This is mainly due to the difficulty of classifying families according to distributions, in most applications, except for simple situations such as those involving only the PK and PU families. The general likelihood formulation is used here as a vehicle to further the understanding of potential problems in using the two-parameter admixture approach. A practical solution that embeds the correct likelihood formulation in a Bayesian framework is being pursued in a separate study.

## Authors' contributions

Both authors were involved in all aspects of this research project. Both authors read and approved the final manuscript.
